# Mother’s Loneliness: Involuntary Separation of Pregnant Women in Maternity Care Settings and Its Effects on the Experience of Mothers during the COVID-19 Pandemic

**DOI:** 10.3390/ijerph19095081

**Published:** 2022-04-21

**Authors:** Paulina Malarkiewicz, Stanisław Maksymowicz, Maria Libura

**Affiliations:** 1 Department of Obstetrics and Gynaecology, School of Medicine, Collegium Medicum of the University of Warmia and Mazury, al. Warszawska 30, 10-082 Olsztyn, Poland; 2 Department of Psychology and Sociology of Health and Public Health, School of Public Health, Collegium Medicum of the University of Warmia and Mazury, al. Warszawska 30, 10-082 Olsztyn, Poland; stanislaw.maksymowicz@uwm.edu.pl; 3 Medical Education and Simulation Department, School of Medicine, Collegium Medicum of the University of Warmia and Mazury, al. Warszawska 30, 10-082 Olsztyn, Poland; maria.libura@uwm.edu.pl

**Keywords:** pregnancy, separation, anxiety, COVID-19, quality of life, maternity care

## Abstract

The aim of the study was to investigate the challenges of involuntary separation experienced by women during pregnancy and childbirth in the time of the COVID-19 pandemic. The study was conducted by the means of a self-administered questionnaire. One thousand and eleven women (1011) from Poland took part in the study, with an average age of approximately 30 years. The study was approved by the Research Ethics Committee of Warmia and Mazury University in Olsztyn, Poland. The results show that the majority of the surveyed women experienced involuntary separation from their partners during pregnancy and childbirth: 66.27% had no choice but to give birth alone and 84.37% had not been able to attend medical appointments with their partners. Solitary encounters with healthcare were associated with the feeling of fear (36.4%), anger (41%), a sense of injustice (52.2%), acute sadness (36.6%) and a sense of loss (42.6%), with all the reported levels higher in younger women. Over 74% of respondents were afraid of childbirth without a partner present. Almost 70% felt depressed because of a lonely delivery experience. Nearly a quarter of the mothers surveyed declared that if they could go back in time, they would not have made the decision to become pregnant during the pandemic. Based on our study, we found that adjustments to prenatal and neonatal care arrangements under COVID-19-related regimens are needed. Our proposal is to implement at least three fundamental actions: (1) risk calculations for pandemic-related cautionary measures should take into account the benefits of the accompanied medical appointments and births, which should be restored and maintained if plausible; (2) medical personnel should be pre-trained to recognise and respond to the needs of patients as a part of crisis preparedness. If the situation does not allow the patient to stay with her family during important moments of maternity care, other forms of contact, including new technologies, should be used; (3) psychological consultation should be available to all patients and their partners. These solutions should be included in the care plan for pregnant women, taking into account a risk-benefit assessment.

## 1. Introduction

Pregnancy is a time of radical changes in the lives of expecting mothers, involving biological, physiological and social functioning spheres. This time is also marked by evolving relationships, especially those with the closest partner/father of the child, beginning with the predominantly mutually agreed decision to become pregnant, throughout the duration of pregnancy itself, to childbirth and postpartum experience. At each of these stages, stress, anxiety and other mental health challenges or even psychiatric disorders may appear. Thus, preparing for the role of a mother and undergoing pregnancy-associated transitions is a possible trigger of psychological disorders, such as antenatal anxiety and depression [1,2]. Often, fears for the success of the pregnancy, the proper development of the fetus and the course of delivery, are also present. One can experience a wide range of emotions during pregnancy, which may often be quite extreme [3].

Mental health disorders become particularly challenging when life and/or health threatening situations occur on a wider social plane, which is undoubtedly the case with the COVID-19 (coronavirus) pandemic that started worldwide in 2020 [4]. Currently, women of a reproductive age do not remember the earlier pandemics of the twentieth centuries, while the more recent ones had a limited impact in terms of social reception in most countries. The global pandemic of severe acute respiratory syndrome (SARS) in 2002–2003 was the first in the 21st century [4]. Yet, since it contained only residents of the threatened areas who were faced with accompanying mental health challenges, the rest of the world were mostly observers. Additionally, the SARS pandemic did not cause as much fear and did not record as many deaths as the COVID-19 pandemic. The swine flu virus epidemic effects were more directly felt in the European region. In the spring of 2009, a new influenza A virus (H1N1) emerged [5]. At first, it was detected in the United States, but soon it spread across the world. H1N1 turned out to be dangerous for pregnant women [6]. It is unofficially estimated that it caused the deaths of approximately 200,000 people [7]. The Ebola epidemic in 2013–2016 did not directly affect European residents [8].

The COVID-19 pandemic can be considered a new and uncertain situation for most of society. No government or healthcare system was prepared for such a crisis. Undoubtedly, the threat to life and/or health associated with a novel infectious disease is a major stress factor. The measures aimed at mitigating the risk of infection themselves may also pose an additional burden on patients. The pandemic also significantly impacted the functioning of the healthcare system, maternity care included. At the beginning of the pandemic in 2020, health officials in Poland suggested that it was advisable to postpone any plans of family enlargement because of potential negative effects of COVID-19 on pregnancy, as well as the limitations in the functioning of hospitals and gynecology clinics. PTGiP (Polish Society of Obstetricians and Gynecologists), following the international guidelines [9], recommended to suspend family births on 20 March 2020. The possibility of accompanying the partner during the ultrasound examination of the pregnant woman was also suspended [10]. However, some pregnancies were already underway, not to mention the fact that it is impossible to suspend reproduction in the entire population. Therefore, the prenatal care had to function in reality of shifting guidelines and changing pandemic restrictions.

The aim of our study was to analyze how the COVID-19 pandemic influenced the quality of life of pregnant women, how it affected their, and their partners, well-being and whether it translates into their future procreative plans.

The pivotal theme of the study is loneliness, resulting from lonely doctor visits and childbirth. Cacioppo et al. defines loneliness as “subjective distress resulting from a discrepancy between desired and perceived social relationships” [11]. Jeste et al. in JAMA Psychiatry suggests that loneliness is a modern epidemic, resulting in serious health disorders [12].

In our study, loneliness turned out to be the dominant emotion during the initial conversations with both pregnant women and women who gave birth to a child under sanitary restrictions, which one of the authors of this study conducted in her medical practice. Medical interviews, which practically piloted the main study, were filled with patients’ complaints about exactly this issue, showing how important this problem was to women and that it requires systematic scientific investigation.

We also wanted to present recommendations aimed at improved focus on mental well-being in maternity care for possible subsequent waves of COVID-19 or future pandemics. The main hypothesis of the present study is the following: pregnant women experienced elevated levels of loneliness linked to the coronavirus pandemic and related sanitary measures, which had a significant impact on their quality of life [13] and procreative plans (planned family expansion).

## 2. Materials and Methods

The study, approved by the Research Ethics Committee of Warmia and Mazury University in Olsztyn, Poland (No. 6/2021), was carried out from January to February 2021, during the second wave of the coronavirus pandemic in Poland. The conditions for participation in the study were: adult age, ongoing pregnancy or delivery during the COVID-19 pandemic and informed consent to participate in the study.

The study was conducted using a self-report questionnaire (on-line and paper), consisting of 44 questions: 42 closed, single-choice and single-choice Likert scale questions and 2 open-questions, divided into 5 domains: (1) demographic data (6 questions), (2) patient’s health status (9 questions), (3) patient’s social standing during pregnancy and childbirth (6 questions), (4) patient’s emotions (17 questions) and (5) the impact of the health care system functioning during a pandemic on the patient’s experiences of pregnancy and childbirth (6 questions).

The questionnaires were disseminated via the Internet and at one maternity hospital in Olsztyn, Poland. The online survey tool was created using a commonly utilized platform, https://docs.google.com/ (accessed on 19 April 2022), which allows respondents to anonymously complete a survey on both mobile devices and standard computers. Investigators used the snowball sampling method to deliver the survey invitation letter with the survey URL to potential participants via the Instagram account, Facebook and email. Participants were invited to forward the survey invitation letter with the survey link to their friends and relatives. All individuals were freely participating in the study and had the option to discontinue at any moment. The survey was anonymous and confidential, and it took about 10 min to complete.

One thousand and eleven women (1011) took part in the study, aged from 17 to 50. A total of 899 participants filled the online form, while one hundred twelve (112) completed the pen and paper version. The average age was 29.5 years. The participants had a higher education degree equivalent to the Master’s level (59.1%, *n* = 597), secondary education (21.8%, *n* = 235) or a Bachelor’s degree (16.5%, *n* = 166). A total of 82.3% (*n* = 832) of the respondents indicated that they were currently employed, 14.8% (*n* = 150) were unemployed and 2.9% (*n* = 29) were studying. The distribution of the place of residence variable was relatively equal, with a slight predominance of villages and large cities. Most of the respondents remained in a permanent formal relationship (77.7%, *n* = 786), with 20.7% (*n* = 209) declaring an informal relationship status.

A total of 49.9% (*n* = 504) of respondents gave birth during the pandemic, before participating in the survey. The remaining women (50.1%, *n* = 507) were pregnant during the pandemic (they were awaiting delivery at the time of filling in the questionnaire). The structure of the study group is presented in Table 1.

### Statistical Analysis and Variable Measurement

Microsoft Excel and IBM SPSS Statistics 27 (Armonk, NY, USA: IBM Corp) were used to perform the analysis. To present the overall characteristics of the data, descriptive statistics for all study variables were calculated. Then, the Pearson’s correlation coefficients and multivariate linear regression analysis were calculated to determine covariates. A two-tailed significance level of 0.05 was used in all statistical tests. The odds ratio was calculated with MedCalc (https://www.medcalc.org/calc/odds_ratio.php (accessed on 5 April 2022).

The internal consistency of the scales was computed using Cronbach’s alpha coefficient. The internal consistency of the items in this study are the following: for the Likert scale questions about emotions related to separation, Cronbach’s alpha is 0.904 (excellent); for the Likert scale questions about women’s emotions, Cronbach’s alpha is 0.804 for pregnant women (acceptable); while for those who gave birth, Cronbach’s alpha is 0.764 (acceptable) [14].

**Figure 1:**
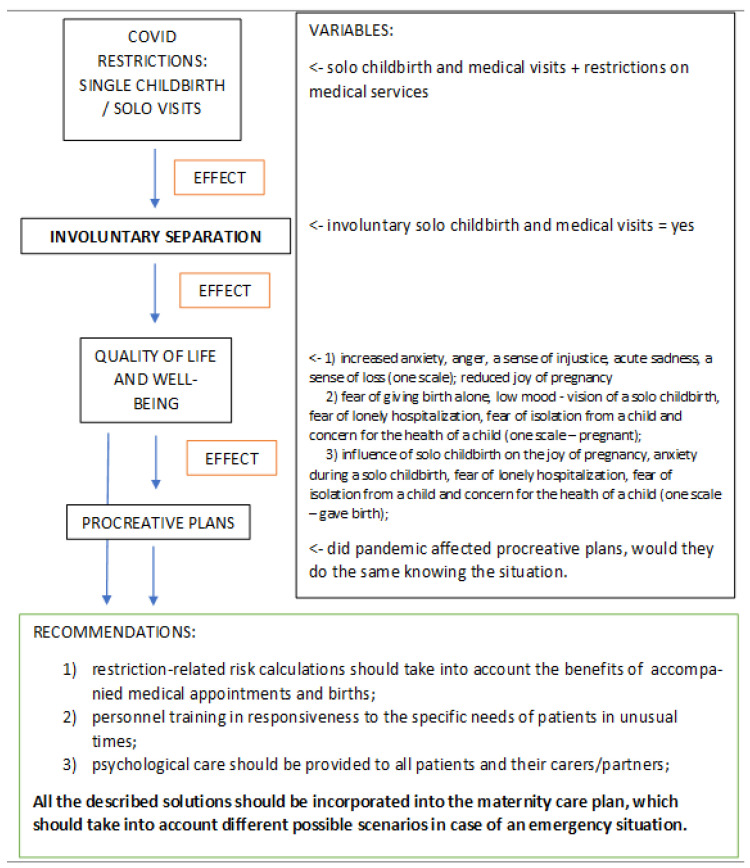
Involuntary separation-measured variables, income and outcome.

The problem of involuntary separation was measured through independent variables: medical visits and delivery status (single or not). The effect of the involuntary separation on quality of life and well-being was measured through dependent variables: (1) increased anxiety, anger, a sense of injustice, acute sadness, a sense of loss (one scale); reduced joy of pregnancy; (2) fear of giving birth alone, low mood regarding the vision of a solo childbirth, fear of lonely hospitalization, fear of isolation from the baby and concern for the health of the baby (one scale—pregnant); (3) influence of solo childbirth on the joy of pregnancy, anxiety during a solo childbirth, fear of lonely hospitalization, fear of isolation from the baby and concern for the health of the baby (one scale—gave birth) (see Figure 1).

## 3. Results

### 3.1. Separation and Emotions

The main hypothesis of the present study is that pregnant women would experience elevated levels of loneliness linked to the coronavirus pandemic and related sanitary measures, which would have a significant impact on their quality of life, was confirmed. The most important limitation for pregnant women was the inability of the partner to participate in prenatal care appointments and be present during delivery. We called this phenomenon, involuntary separation. This term means that the separation of the patient from her closest social circle is imposed rather than chosen. The way in which the problem was measured and its impact on the quality of life, well-being, procreative plans as well as the resulting recommendations, are shown in Figure 1.

Among all the women surveyed, 84.37% (*n* = 853) admitted that during pregnancy, they had not been able to attend medical appointments with their partners. Respondents who gave birth during the pandemic, in 66.27% (*n* = 334) of cases, had to do it alone due to pandemic-related measures. Only for 7.34% (*n* = 37), it was a chosen option. Furthermore, 26.39% (*n* = 133) were allowed to have a husband/partner by their side at the time of delivery. As a result of multiple regression analysis with Age, Education, Occupational status, Place of residence size and Marital status as independent variables and Partner’s participation in medical appointments as a dependent variable, one can conclude that there were no significant differences in any of the variables.

Respondents indicated that due to lonely doctor appointments, they experienced increased anxiety (36.4%, *n* = 368), anger (41%, *n* = 415), a sense of injustice (52.2%, *n* = 527), acute sadness (36.6%, *n* = 369) and a sense of loss (42.6%, *n* = 431). This problem affected both groups of respondents (pregnant women and those who had already given birth at the time of questionnaire completion) equally, as both were limited by the systemic restrictions.

The impact of separation shown as a series of negative emotions proved to be strongly associated with some demographic variables. The age of the patient was significantly negatively associated with all negative experiences. This means that the younger the women, the higher the index of negative emotions accompanying the separation. Another age-related variable was the level of education, which again was strongly associated with the experience of anger, a sense of injustice and overwhelming sadness. Marital status was not as strongly associated with the emotions of anger and an overwhelming sadness. The other measured demographic variables, such as occupational status and place of residence size, were not statistically significantly associated with negative emotions (Table 2A).

The multivariate linear regression analysis of independent variables (demographic: Age, Education, Occupational status, Place of residence and Marital status) and dependent variables (Increased anxiety, Anger, Feeling of injustice, Overwhelming sadness and a Sense of loss) confirmed that there is a strong association between dependent variables and a younger age (Beta for Increased anxiety is −0.126, for Anger is −0.176, for Feeling of injustice is −0.171, for Overwhelming sadness is −0.147 and for a Sense of loss is −0.166; all variables are significantly associated). Anger was also significantly associated with Education (Beta is −0.102). Multivariate linear regression analysis and odds ratio are shown in Table 2B. The detailed results of the regression analysis are in the Supplement file: Table S1–S5.

The odds ratio for independent variable, Age, and Increased anxiety is 1.54 (1.7 to 2.04, *p* = 0.0021), Anger is 1.8 (1.4 to 2.4, *p* < 0.0001), Feeling of injustice is 1.6 (1.2 to 2.2, *p* = 0.0009), Overwhelming sadness is 1.8 (1.4 to 2.4, *p* < 0.0001) and a Sense of loss is 1.18 (0.9 to 1.5, *p* = 0.023, insignificant). The odds ratio was also significant for Education and Anger, and is 1.77 (1.28 to 2.45, *p* = 0.0005). Thus, it was confirmed that the problems related to separation concern young women to a greater extent. Other variables are not significantly associated (Table 2B).

For the further part of the analysis, our sample was divided into two groups: (1) women who were pregnant during the pandemic (at the time they completed the questionnaire) and (2) women who gave birth during the pandemic (before they completed the questionnaire).

Our assumption was that the experiences of these groups, although similar, will differ on selected planes. Both groups indicated that the loneliness they experienced in maternity care affected their overall well-being, and might also have far-reaching effects.

#### 3.1.1. Women Who Were Pregnant during the Pandemic

The majority of women in this group admitted that they were afraid of a lonely childbirth (74.1%, *n* = 376, of which 55.4%, *n* = 281 were definitely afraid of such a situation). For 69.6% (*n* = 353), this perspective was a cause of a lowered mood, and this applied to all women regardless of demographic characteristics or other tested variables.

**Figure 2:**
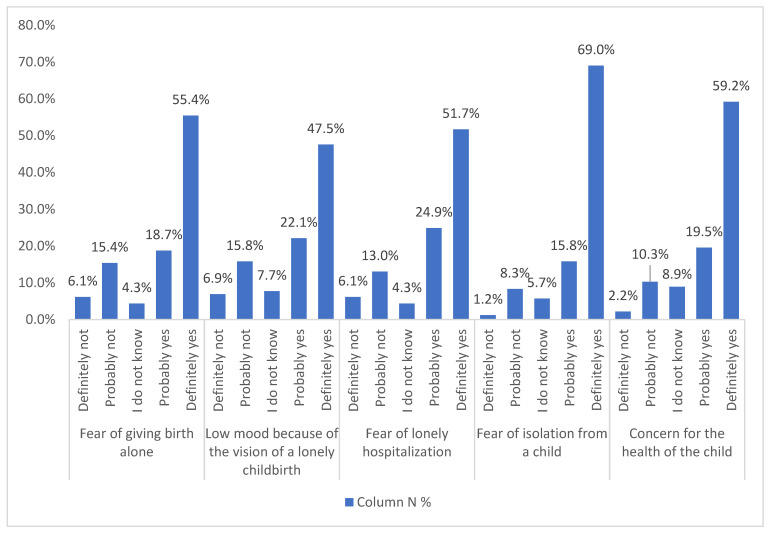
Emotions related with pregnancy (*n* = 507).

Even more pregnant women were afraid of a lonely hospital stay (76.6%, *n* = 388). This worry was statistically significantly (Pearson correlation) associated with a younger age (*p* < 0.002), but also with the lower level of education (*p* < 0.023) and occupational status (*p* < 0.047). A total of 84.8% (*n* = 430) of women in this group were also concerned with the prospect of isolation from their child after childbirth (without significant demographic differences). Slightly less, 78.7% (*n* = 399), were anxious about their child’s health (variable significantly related to marital status, *p* < 0.022). The above data are illustrated in Figure 2.

#### 3.1.2. Women Who Gave Birth during the Pandemic

Such fears of pregnant woman were mirrored by the experiences of those who gave birth during the pandemic. First of all, a large proportion of respondents in this group (62.9%, *n* = 317) declared that the absence of a partner during childbirth negatively impacted their level of happiness. This reduction was statistically significantly (Pearson correlation) associated with occupational status (*p* < 0.008), the course of childbirth (i.e., giving birth alone, *p* < 0.015) and partner’s participation in earlier medical appointments (associating with lonely medical appointments, *p* < 0.016).

**Figure 3:**
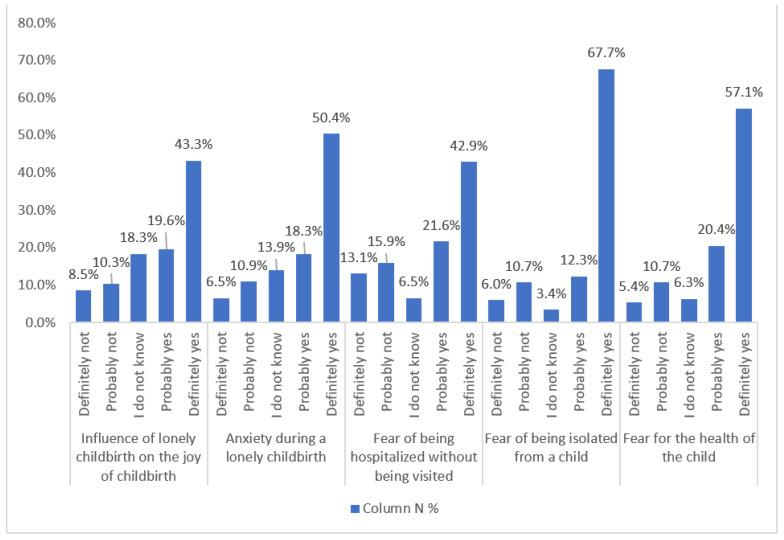
Emotions related with childbirth (*n* = 504).

A higher level of anxiety (reported by 68.7% of participants, *n* = 346) was statistically significantly (Pearson correlation) associated with a lower age (*p* < 0.002) and a lack of partner’s participation in medical appointments (*p* < 0.030). Women giving birth during the pandemic also admitted that they were afraid of loneliness during hospitalization, resulting from visiting policies (64.5%, *n* = 325); this variable was significantly associated with a lower age (*p* < 0.002). Many women experienced the fear of being separated from their child (80%, *n* = 403), another variable significantly associated with a lower age at the *p* level of <0.011 (even though only about 5% were actually separated from the child), as well as fears concerning their infant’s health (77.5%, *n* = 391, without any statistically significant association with demographic and other variables). The above data are illustrated in Figure 3.

### 3.2. Restrictions and Healthcare System

We also examined how the pandemic restrictions influenced the experiences related to pregnancy in general, and the encounters with the healthcare system’s services in particular. Almost a third of the women surveyed admitted that the pandemic restrictions reduced their happiness during pregnancy (29%, *n* = 293 of the respondents indicated “definitely yes” or “probably yes”; 50.5%, *n* = 511 indicated “probably not” and “definitely not”; 20.5%, *n* = 207 do “not know”; a statistically significant association with demographic variables was found only with the place of residence variable (*p* < 0.005, Pearson correlation), and it was stronger in smaller towns and villages).

One of the limitations mentioned in the questionnaire was related to the availability of maternity care services, especially the restricted access to medical examinations. A total of 18% (*n* = 182) of the respondents indicated that due to the pandemic, they missed some prenatal care appointments, between two or three on average. Due to the restrictions, 17% (*n* = 172) experienced a limited access to health services, including tests and vaccinations. Among the most frequently indicated were: vaccinations, OGTT 75 g (Oral Glucose Tolerance Test), 11 to 13 + 6 scan (first trimester screening), second trimester anomaly scan and amniocentesis.

In extreme cases, as some women wrote in the survey, no tests were performed other than one ultrasound. Access to basic tests such as blood count and urinalysis was also difficult. One of the respondents wrote in a comment that some of the appointments took the form of teleconsultations (one may suspect that these were relatively frequent, but we did not ask the respondents directly).

Respondents were asked if their pregnancy care was altered by the pandemic: 18% (*n* = 182) indicated that the care quality worsened, 49.2% (*n* = 497) declared it did not change, while for 1.6% (*n* = 16), it improved, with 31.3% (*n* = 316) expressing no opinion. Thus, taking into account both restricted access and perceived care quality, nearly 1/5 of respondents experienced lower care quality and/or accessibility due to the pandemic, regardless of demographic characteristics.

As many as a quarter of the respondents (23.9%, *n* = 242) declared that they would have waited until the end of the pandemic before getting pregnant if they had known what was awaiting them. However, when asked whether the pandemic influenced their decision to get pregnant, the majority of women (78.3%, *n* = 792) admitted that it did not influence their decision (Table 3).

Moreover, participants in our study suffered from restricted access to information related to childbirth since the onset of pandemic. A total of 40.4% (*n* = 408) of the women said that they did not receive any information. A total of 24.8% (*n* = 251) said that the information they received was insufficient. Relations with healthcare personnel were also affected, with more than 1/5 of the respondents (21.2%, *n* = 214) declaring deterioration in this respect. Detailed results of the measurement of the effect of epidemic restrictions are presented in Table 3.

## 4. Discussion

Pregnancy involves a complex psychological process [15]. The fertility of a woman, her age, socioeconomic status and family situation are important factors [16]. The course of pregnancy involving complications also increases the incidence of depression in pregnant women. The context of the pandemic had a significant impact on the mental well-being of our respondents, which is in line with other research; in the systematic review of the prevalence of anxiety during the COVID-19 pandemic, it proved to be significantly higher among females, especially in Europe [17].

Anxiety is the most common mental health issue present during pregnancy. Rauf et al. found that during the pandemic, women experienced increased perinatal anxiety, and a lower availability of appropriate obstetric healthcare, which we also observed in our study. Moreover, in Rauf et al.’s study, women experienced increased levels of fear for their own health, as well as that of their baby’s, which was also an important aspect in our study [18]. Dunkel Shetter et al. emphasized that anxiety and stress during pregnancy are risk factors for negative outcomes for both mothers and children. Depression and post-traumatic stress disorder most often concern the perinatal period, i.e., 4–6 weeks after delivery [19]. As shown in a recent study, increased levels of anxiety scores were strongly associated with SARS-CoV-2 infection during pregnancy [20].

Due to the forced separation from the father of the child, some needs and expectations of women during pregnancy and childbirth were compromised. The patients mainly regretted limited participation of their partners in maternity care appointments and their absence during delivery. Before the outbreak of the pandemic, patients were able to freely use medical care in the company of a life partner. As showed by Cheng et al., the role of a partner in the care for a pregnant woman is crucial. Among others, low partner support was associated with higher pregnancy-related anxiety [21]. However, in our study, the lack of partner’s support was not intended but forced by sanitary restrictions (involuntary separation). Perhaps, for this reason, the absence of a partner during antenatal care generated the sense of injustice and anger among our responders. The systematic review of Antoniou et al. established that a correlation between the support from the partner and prenatal mental disorders and anxiety in women was common [22].

This is in concord with a previous study, showing that the presence of a partner during labor increases positive birth experiences. The partner provides support and assistance to the pregnant woman during childbirth, reducing her anxiety. The presence of a known and trusted person in the delivery room is essential for most first-time delivery patients [23].

Finally, research shows that when crises are affecting society at large, support and planning are needed for pregnant women, who are a particularly vulnerable [24]. What we propose as the conclusions of our study, is a list of recommendations to such effect.

## 5. Conclusions

The pandemic turned out to have a significant impact on the quality of life and experiences of pregnant women who participated in our study, especially those of a younger age. During pregnancy, higher levels of anxiety and stress are observed. Among our respondents, the pandemic significantly influenced both, especially due to the perspective of a lonely childbirth and the risk of separation from an infant and its father/partner. It was a new and unpredictable situation that markedly influenced the well-being of pregnant women in all its dimensions: psychological, sociological and even physical. Being pregnant and giving birth to a child is a pivotal life event. Contrary to expectations, pregnant patients were denied access to prenatal care in the partner’s company, and family births and appointments were often suspended. Perinatal loneliness was not a choice, but a necessity.

Involuntary separation that occurs widely [25] is a new sociological phenomenon, not only for pregnant women, but also for all patients at the time of the current pandemic. Despite many restrictions, 50.5% (*n* = 511) of the respondents declared that it did not diminish their joy of pregnancy. In quite important moments, such as the obstetric appointment or delivery, the partner could not take part. As the role of the partner appears to be very important during pregnancy, this forced absence significantly increased the feeling of anxiety. Pregnant women also struggled with a high sense of injustice. However, the decision to get pregnant was seemingly not influenced by the pandemic, with only 8.4% (*n* = 85) of the respondents declaring it had an impact on their decision. However, this artefact can be explained by the fact that half of the respondents gave birth during the early stage of the pandemic (49.9%, *n* = 504); thus, the decision to conceive must have preceded its onset.

The fear of contracting COVID-19 during pregnancy (61%, *n* = 617) and the importance of having a partner present during medical appointments and delivery were highlighted as main challenges. The experiences of the patients we examined have shown that there is still no answer to the loneliness problem in the health care system. Unfortunately, these experiences may have a significant impact on subsequent procreation plans.

While writing this article, we are currently in the middle of the fifth wave of the COVID-19 pandemic. Appointments with a partner are still banned in many places, but more and more people have already been vaccinated against COVID-19. The situation of pregnant women is improving. However, it does not change the fact that patients who have already given birth feel a sense of loss. We hope that with more knowledge about the new SARS-CoV-2 virus and the increasing number of vaccinated people, we will be able to gradually return to the standards of perinatal care from before the outbreak of the pandemic.

### Recommendations

Based on our research, we would like to present recommendations that can contribute to better prenatal care for pregnant women and their partners in the current and future pandemics:

Firstly, restriction-related risk calculations should take into account the benefits of accompanied medical appointments and births, which, if possible, should be restored and maintained. These actions can reduce the burden of stress during pregnancy and its consequences. The presence of a partner during pregnancy and childbirth is often paramount for a woman. Additionally, although the partner’s participation may pose an additional risk of infection, available verification methods, e.g., by rapid antigen tests or PCR tests, should be used instead of a top-down ban on joint visits.

Secondly, as shown by research and clinical experience, special situations require special preparation of personnel. It can be achieved through training in response to the specific needs of patients in unusual times. If the situation does not actually allow the patient to contact her family, other forms of contact and new technologies should be used, e.g., video calls, remote or hybrid participation.

Thirdly, psychological care should be provided to all patients and their carers/partners. Psychological consultation should be offered to each patient and her partner. This is especially true of young pregnant women and those giving birth for the first time, and women who are less well-off. This could help identify patients requiring therapy or psychiatric treatment. Moreover, offering patients as much support and empathy as possible is crucial. Most of the respondents indicated insufficient access to information about labor and hospitalization during the pandemic. It is important to improve communication among health professionals and patients. All the regulations related to the necessary restrictions should be presented in simple terms and explained to patients.

All the described solutions should be incorporated into the maternity care plan, which should take into account different possible scenarios in case of an emergency situation.

## 6. Limitations

Due to epidemic restrictions, the study was largely conducted via the Internet. Only a small number of respondents completed the questionnaire at the clinic (*n* = 112). Additionally, the selection of the sample was not random, but based on the snowball method. Even though a relatively heterogeneous group was obtained, a large overrepresentation of women with a higher education was observed. Moreover, the study was not preceded by extensive qualitative piloting (only by the doctor’s conversations with patients and desk-research). We also have not collected data on the exact time since the birth. However, the questionnaire was administered at the turn of January and February 2021, so all respondents had given birth up to 12 months prior (the pandemic started in Poland in March 2020).

## Figures and Tables

**Table 1:** Characteristics of the studied group.

		Count	Column N % (N = 1011)
Age	17–24	110	10.9%
	25–29	423	41.8%
	30–34	347	34.3%
	35–39	114	11.3%
	40–44	15	1.5%
	45–50	2	0.2%
Education	Primary education	13	1.3%
	Secondary education	235	23.2%
	Higher education—Bachelor’s degree	166	16.4%
	Higher education—Master’s degree	597	59.1%
Occupational status	Employed	832	82.3%
	Studying	29	2.9%
	Unemployed	150	14.8%
Place of residence (the number of residents)	Countryside	237	23.4%
	City up to 50,000	208	20.6%
	City 50,000–150,000	131	13.0%
	City 150,000–500,000	196	19.4%
	City over 500,000	239	23.6%
	Single	10	1.0%
	In an informal relationship	209	20.7%
Marital status	Married	786	77.7%
	Divorced	4	0.4%
	Widowed	2	0.2%
Health status (pregnancy and childbirth)	Pregnant during the pandemic (responded before giving birth)	507	50.1%
	Gave birth during the pandemic	504	49.9%
Involuntary separation	Solo medical appointments during pregnancy (among all studied women)	853	84.37%
	Solo childbirth (among women who gave birth)	334	66.27%

**Table 2:** (**A**) Pearson correlation between demographic variables and perceived emotions related to separation. (**B**) Multivariate linear regression analysis and odds ratio.

(A)						
		Age	Education	Occupational Status	Place of Residence Size	Marital Status
Increased anxiety	Pearson correlation	−0.125 **	−0.061	−0.002	0.048	−0.047
	Sig. (two-tailed)	<0.000	0.081	0.963	0.168	0.174
	N	822	822	822	822	822
Anger	Pearson correlation	−0.202 **	−0.149 **	0.013	−0.043	−0.075 *
	Sig. (two-tailed)	<0.000	<0.000	0.719	0.211	0.031
	N	829	829	829	829	829
Feeling of injustice	Pearson correlation	−0.181 **	−0.098 **	0.006	−0.027	−0.049
	Sig. (two-tailed)	<0.000	<0.005	0.854	0.439	0.155
	N	832	832	832	832	832
Overwhelming sadness	Pearson correlation	−0.177 **	−0.138 **	0.034	−0.026	−0.087 *
	Sig. (two-tailed)	<0.000	<0.000	0.335	0.465	0.013
	N	823	823	823	823	823
A sense of loss	Pearson correlation	−0.111 **	−0.056	−0.034	0.056	−0.039
	Sig. (two-tailed)	<0.001	0.106	0.326	0.107	0.258
	N	826	826	826	826	826
**(B)**						
		**Age**	**Education**	**Occupational Status**	**Place of Residence Size**	**Marital Status**
Increased anxiety	Standardized Coefficients Beta	−0.126	−0.038	−0.028	0.070	−0.019
	Sig.	<0.001	0.334	0.445	0.051	0.591
	OR (CI and *p*-value)	1.54 (1.17 to 2.04, *p* = 0.0021)	1.33 (0.97 to 1.84, *p* = 0.0781)	0.92 (0.64 to 1.32, *p* = 0.6646)	0.8 (0.60 to 1.06, *p* = 0.1204)	1.44 (1.03 to 2.03, *p* = 0.0327)
Anger	Standardized Coefficients Beta	−0.176	−0.102	−0.058	−0.002	−0.026
	Sig.	<0.001	0.008	0.108	0.958	0.459
	OR (CI and *p*-value)	1.80 (1.36 to 2.37, *p* < 0.0001)	1.77 (1.28 to 2.45, *p* = 0.0005)	0.83 (0.58 to 1.19, *p* = 0.3200)	1.14 (0.86 to 1.50, *p* = 0.3458)	1.5 (1.07 to 2.12, *p* = 0.0172)
Feeling of injustice	Standardized Coefficients Beta	−0.171	−0.052	−0.045	0.002	−0.012
	Sig.	<0.001	0.172	0.215	0.950	0.734
	OR (CI and *p*-value)	1.62 (1.22 to 2.15, *p* = 0.0009)	1.43 (1.02 to 1.02, *p* = 0.0386)	0.97 (0.66 to 1.41, *p* = 0.8808)	1.07 (0.8 to 1.42, *p* = 0.6278)	1.46 (1.01 to 2.10, *p* = 0.0402)
Overwhelming sadness	Standardized Coefficients Beta	−0.147	−0.090	−0.027	0.013	−0.044
	Sig.	<0.001	0.020	0.467	0.722	0.216
	OR (CI and *p*-value)	1.82 (1.38 to 2.4, *p* < 0.0001)	1.63 (1.18 to 2.24, *p* = 0.0029)	0.83 (0.057 to 1.19, *p* = 0.3229)	1.12 (0.85 to 1.48, *p* = 0.4095)	1.42 (1.0 to 1.98, *p* = 0.0438)
A sense of loss	Standardized Coefficients Beta	−0.116	−0.048	−0.059	0.074	−0.013
	Sig.	0.002	0.218	0.108	0.040	0.723
	OR (CI and *p*-value)	1.18 (0.9 to 1.55, *p* = 0.023)	1.16 (0.84 to 1.6, *p* = 0.3538)	1.16 (0.81 to 1.68, *p* = 0.3975)	0.76 (0.58 to 1.01, *p* = 0.0617)	1.22 (0.87 to 1.71, *p* = 0.2446)

** Correlation is significant at the 0.01 level (two-tailed). * Correlation is significant at the 0.05 level (two-tailed).

**Table 3:** Effects of epidemic restrictions.

		Count	Column N % (N = 1011)
Impact of pandemic restrictions on happiness during pregnancy: reduced happiness	Definitely and probably yes	293	29%
	Definitely and probably not	511	50.5%
	Do not know	207	20.5%
Healthcare availability during pandemic (% does not accumulate—affirmative answers from different questions)	Missed prenatal care appointments	182	18%
	Limited access to health services	172	17%
	Limited access to main doctor	170	16.8%
	Limited access to the hospital emergency ward	275	27.2%
Healthcare quality during pandemic	Worsened	182	18%
	Did not changed	497	49.2%
	Improved	16	1.6%
	Hard to say	316	31.3%
Access to information related to childbirth since the onset of pandemic	Did not received all sufficient information	408	40.4%
	Received, but lacked sufficient information	251	24.8%
	Received all sufficient information	352	34.8%
	Worsened	214	21.2%
Relations with healthcare personnel	Did not changed	709	70.1%
	Improved	88	8.7%
Decision to get pregnant	I would do the same	769	76.1%
	I would not decide to get pregnant	242	23.9%
	No	792	78.3%
Did the pandemic influence the decision to get pregnant?	Yes	85	8.4%
	Hard to say	134	13.3%

## Data Availability

The datasets are available from the corresponding author on reasonable request.

## Supplementary Materials

The following supporting information can be downloaded at: https://www.mdpi.com/article/10.3390/ijerph19095081/s1, Table S1. Multivariate linear regression analysis: increased anxiety, Table S2. Multivariate linear regression analysis: anger, Table S3. Multivariate linear regression analysis: feeling of injustice, Table S4. Multivariate linear regression analysis: overwhelming sadness, Table S5. Multivariate linear regression analysis: a sense of loss.
