# Supplementary material for: Mother’s Loneliness: Involuntary Separation of Pregnant Women in Maternity Care Settings and Its Effects on the Experience of Mothers during the COVID-19 Pandemic
Source: Int J Environ Res Public Health. 2022 Apr 21;19(9):5081. doi: 10.3390/ijerph19095081 (PMC9099559; doi:10.3390/ijerph19095081)
Supplement: Supplementary file 1 [file ijerph-19-05081-s001.zip › ijerph-1636424-supplement.pdf]

Supplement

Table S1. Multivariate linear regression analysis: increased anxiety.

Coefficients[a]

Model		Unstandardized Coefficients		Standardized Coefficients		
		B	Std. Error	Beta	t	Sig.
1	(Constant)	3,812	,411		9,270	<,001
	Age	-,215	,063	-,126	-3,388	<,001
	Education	-,065	,067	-,038	-,967	,334
	Occupational status	-,059	,078	-,028	-,764	,445
	Place of residence size	,069	,035	,070	1,952	,051
	Marital status	-,066	,123	-,019	-,538	,591

a. Dependent Variable: increased anxiety

Table S2. Multivariate linear regression analysis: anger.

Coefficients[a]

Model		Unstandardized Coefficients		Standardized Coefficients		
		B	Std. Error	Beta	t	Sig.
1	(Constant)	4,961	,413		12,004	<,001
	Age	-,303	,063	-,176	-4,819	<,001
	Education	-,180	,067	-,102	-2,671	,008
	Occupational status	-,125	,078	-,058	-1,610	,108
	Place of residence size	-,002	,036	-,002	-,053	,958
	Marital status	-,091	,123	-,026	-,741	,459

a. Dependent Variable: Anger

Table S3. Multivariate linear regression analysis: feeling of injustice.

Coefficients[a]

Model		Unstandardized Coefficients		Standardized Coefficients		
		B	Std. Error	Beta	t	Sig.
1	(Constant)	4,803	,401		11,982	<,001
	Age	-,287	,061	-,171	-4,674	<,001
	Education	-,089	,065	-,052	-1,366	,172
	Occupational status	-,093	,075	-,045	-1,242	,215
	Place of residence size	,002	,034	,002	,063	,950
	Marital status	-,040	,119	-,012	-,340	,734

a. Dependent Variable: Feeling of injustice

Table S4. Multivariate linear regression analysis: overwhelming sadness.

Coefficients[a]

Model		Unstandardized Coefficients		Standardized Coefficients		
		B	Std. Error	Beta	t	Sig.
1	(Constant)	4,561	,400		11,400	<,001
	Age	-,243	,061	-,147	-4,004	<,001
	Education	-,151	,065	-,090	-2,329	,020
	Occupational status	-,055	,075	-,027	-,727	,467
	Place of residence size	,012	,034	,013	,356	,722
	Marital status	-,147	,119	-,044	-1,237	,216

a. Dependent Variable: An overwhelming sadness

Table S5. Multivariate linear regression analysis: a sense of loss.

Coefficients[a]

Model		Unstandardized Coefficients		Standardized Coefficients		
		B	Std. Error	Beta	t	Sig.
1	(Constant)	4,076	,410		9,948	<,001
	Age	-,199	,063	-,116	-3,142	,002
	Education	-,082	,067	-,048	-1,232	,218
	Occupational status	-,124	,077	-,059	-1,610	,108
	Place of residence size	,073	,035	,074	2,061	,040
	Marital status	-,043	,123	-,013	-,354	,723

a. Dependent Variable: A sense of loss
